# Field Evaluation of Culture plus Latex Sweep Serotyping for Detection of Multiple Pneumococcal Serotype Colonisation in Infants and Young Children

**DOI:** 10.1371/journal.pone.0067933

**Published:** 2013-07-02

**Authors:** Paul Turner, Claudia Turner, Auscharee Jankhot, Kawalee Phakaudom, Francois Nosten, David Goldblatt

**Affiliations:** 1 Shoklo Malaria Research Unit, Mae Sot, Thailand; 2 Mahidol-Oxford Tropical Medicine Research Unit, Bangkok, Thailand; 3 Centre for Tropical Medicine, University of Oxford, Oxford, United Kingdom; 4 Immunobiology Unit, Institute of Child Health, University College London, London, United Kingdom; Centers for Disease Control & Prevention, United States of America

## Abstract

**Background:**

Nasopharyngeal swab (NPS) culture by World Health Organisation (WHO) methodology underestimates multiple pneumococcal serotype colonisation compared to a simple culture and latex sweep method. The impacts of this on descriptions of pneumococcal serotype distributions and colonisation dynamics in infancy are not clear.

**Methods:**

8,736 NPS collected from infants enrolled into a longitudinal study were processed to evaluate the field utility of the latex sweep method. 1,107 had previously been cultured by WHO methodology. Additionally, colonisation results were compared in 100 matched pairs of infants, where swabs from an individual were cultured either by WHO or latex sweep method.

**Results:**

In 1,107 swabs cultured by both methods, the latex sweep method was three times more likely to detect colonisation with multiple pneumococcal serotypes than the WHO method (p<0.001). At least one common serotype was identified in 91.2% of swabs from which typeable pneumococci were detected by both methods. Agreement improved with increasing colonisation density (p = 0.03). Estimates of age at first pneumococcal acquisition and colonisation duration were not affected by culture/serotyping method. However, a greater number of serotype carriage episodes were detected in infants cultured by latex sweep (p = 0.03). The overall rate of non-vaccine type pneumococcal acquisition was also greater in infants cultured by latex sweep (p = 0.04).

**Conclusions:**

Latex sweep serotyping was feasible to perform on a large specimen collection. Multiple serotype colonisation detection was significantly improved compared with WHO methodology. However, use of the latex sweep method is unlikely to significantly alter colonisation study serotype distribution or colonisation dynamics results.

## Introduction

*Streptococcus pneumoniae* is a common inhabitant of the human nasopharynx and colonisation is thought to always precede infection [1]. Understanding the dynamics of colonisation is of considerable importance now that pneumococcal conjugate vaccines (PCV), covering 7–13 of the >90 pneumococcal serotypes, have been introduced into many countries routine childhood immunisation schedules. These vaccines reduce colonisation by serotypes included in the formulation [2]. As a result of this, there have been observed changes in the pneumococcal serotypes associated with colonisation and invasive disease [3], [4].

We have previously demonstrated that the standard WHO culture and serotyping method for processing nasopharyngeal swabs (NPS), as described in [5], is an inadequate tool for detecting and characterising multiple serotype colonisation. Following culture of NPS specimens on selective agar plates (5% sheep blood with colistin and nalidixic acid (CNA)), both serotyping a sweep of colonies using homemade latex reagents or molecular serotyping by microarray identified significantly more instances of multiple serotype colonisation compared to selective culture followed by serotyping colonies based on morphological differences [6]. This finding raised questions regarding studies of pneumococcal colonisation dynamics:

- Do methodological differences significantly influence reported pneumococcal serotype distributions?
- Is there a methodological effect on estimates of serotype acquisition rates and carriage duration?

The objectives of the current study were to perform selective culture plus latex sweep serotyping on NPS specimens collected during a SE Asian birth cohort study and to subsequently compare pneumococcal colonisation results both within infants (WHO method versus latex sweep method) and between groups of infants (NPS from one group assessed by the WHO method and the second group by the latex sweep method).

## Methods

### Study Population and Specimen Collection

**Figure 1:**
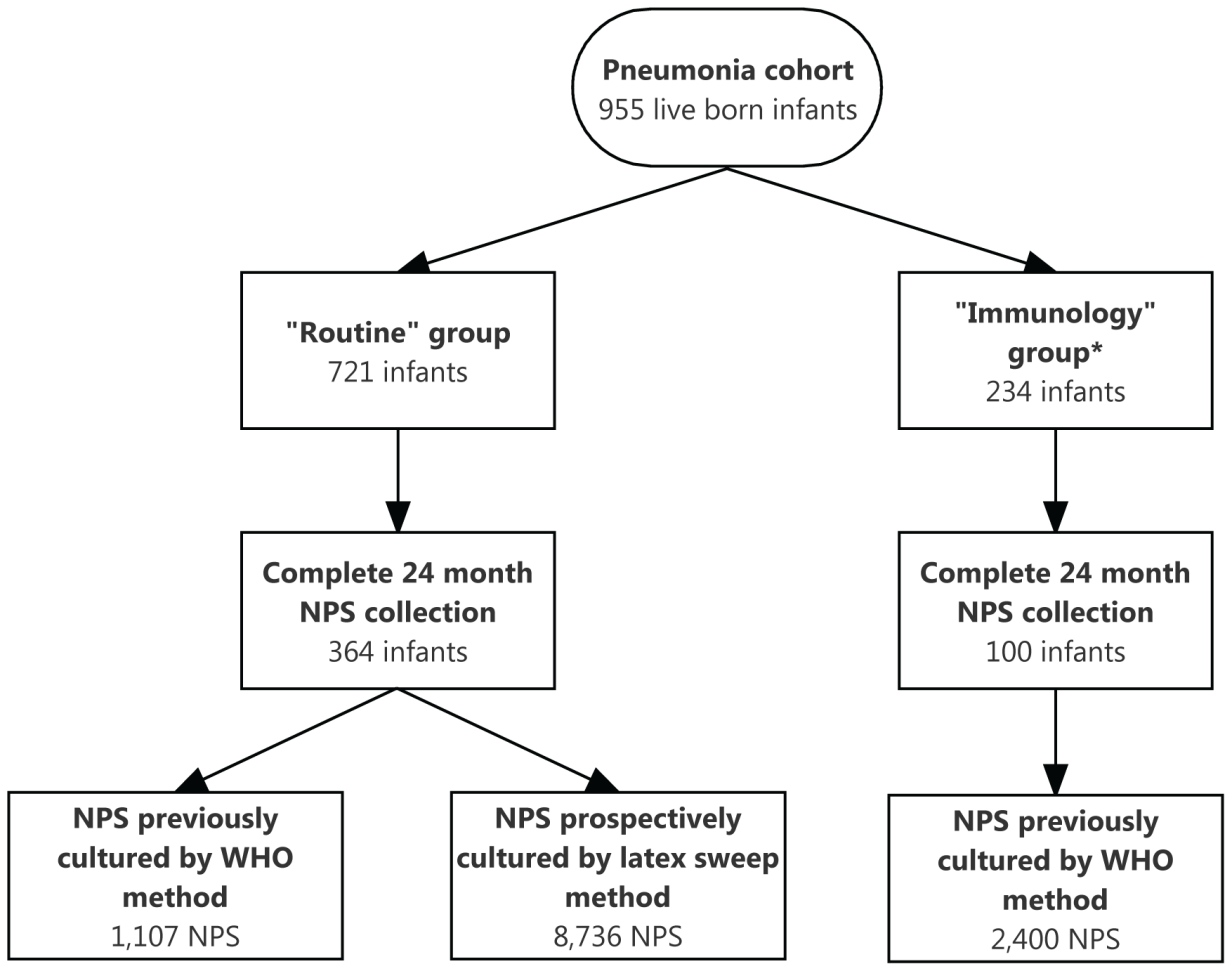
Study flow diagram. * Described in detail in reference [8].

Monthly nasopharyngeal swab (NPS) specimens were collected from 955 infants between one and 24 months of age as part of a longitudinal cohort study of pneumonia epidemiology in Maela camp for displaced persons on the Thailand-Myanmar border [7]. Two hundred and thirty four infants and their mothers were randomly selected to be also included in a detailed pneumococcal carriage study (the “immunology” follow-up group) described elsewhere [8]. The remaining 721 infants formed the “routine” follow-up group (Figure 1). Pneumococcal vaccines were not available in the study population.

The WHO protocol for specimen collection was followed [5]. Briefly, Dacron-tipped flexible swabs (Medical Wire & Equipment, Corsham, UK) were used to sample the nasopharynx. Swab tips were immediately removed into sterile cryovials (Simport, Beloeil QC, Canada) containing 1mL STGG medium (skim milk-tryptone-glucose-glycerol medium; prepared in house) using 70% ethanol-cleaned scissors. These NPS-STGG specimens were transported to the laboratory in a cool box where they were briefly vortexed and subsequently frozen at -80°C within eight hours of collection.

### Specimen Selection, Culture, and Pneumococcal Serotyping

On completion of the study follow-up, the specimen database was reviewed and all NPS from infants in the “routine” follow-up group, in whom a complete set of 24 monthly specimens had been obtained, were selected for assessment by the latex sweep method. A proportion of these NPS specimens had previously been cultured according to the WHO method (Figure 1). Laboratory technicians performing the latex sweep serotyping were blinded to the WHO culture results.

To compare pneumococcal acquisition rates and carriage durations by culture/serotyping method, a subset of infants were studied further. Infants from the “immunology” follow-up group (NPS previously processed by the WHO method and described in detail in [8]) were matched 1∶1 by gender and month of birth with a “routine” follow-up group infant (NPS prospectively processed by the latex sweep method) using the “vmatch” command in Stata/IC 12.1 (StataCorp, College Station TX, USA).

For the latex sweep method, 10 µL fully thawed NPS-STGG was streaked onto a 5% sheep blood-CNA agar plate (bioMerieux, Marcy L’Etoile, France) and incubated overnight at 36°C in 5% CO_2_. All growth from the agar plate were picked using a sterile cotton swab (Medical Wire & Equipment, Corsham, UK) and suspended in sterile 0.85% saline to a density of at least 0.5 McFarland. This suspension was used immediately for serotyping using latex reagents prepared in-house [9], [10]. The suspension was first tested with antisera pools A-I and subsequently with all appropriate group, type, and factor antisera.

For the WHO method, 10 µL of fully thawed NPS-STGG was cultured as described above. All morphologically distinct alpha-haemolytic colonies were sub-cultured onto 5% sheep blood agar (Clinical Diagnostics, Bangkok, Thailand) *S. pneumoniae* was identified by colony morphology, Gram’s stain, and optochin disc susceptibility (Oxoid, Basingstoke, UK) [5]. Isolates with intermediate susceptibility to optochin, defined as a inhibition zone diameter of 7–13 mm, were subjected to tube bile solubility testing [11]. Isolates were serotyped by latex agglutination, using the same in-house reagents as used for the latex sweep method, with Quellung confirmation of equivocal results [9], [10], [12].

For both methods, colonisation density was assessed by estimating the number of pneumococcal colonies in each of the four agar plate quadrants: 1+ (growth in quadrant 1 (inoculation area/primary streak) but <10 colonies in quadrant 2), 2+ (>10 colonies in quadrant 2 but <10 colonies in quadrant 3), 3+ (>10 colonies in quadrant 3 but <10 colonies in quadrant 4), or 4+ (>10 colonies in quadrant 4).

### Definitions of Carriage

The definitions of serotype acquisition, clearance, and duration employed were those used in the study of pneumococcal colonisation mother-infant pairs [8]. Briefly, acquisition was defined as the first appearance of a pneumococcal serotype in an infant’s NPS culture or when a serotype was re-cultured following clearance. Clearance was defined as the absence of a serotype from two consecutive NPS specimens. Serotype carriage episode duration was calculated as the interval between acquisition (mid-point between last negative and first positive swab) and clearance (mid-point between last positive and first of two consecutive negative swabs).

### Data Analysis

Data analyses were performed using Stata/IC 12.1. Comparisons of serotype detections by WHO culture or latex sweep serotyping were made by McNemar’s test. Continuous numerical variables were summarised by means and groups were compared by t-test. Serotype acquisition rates and carriage durations were calculated by survival analysis; groups were compared using the log-rank test.

Serotypes 15B and 15C were considered to be a single serotype (15B/C) given the lability of their capsular polysaccharides [13]. Serotypes 6A and 6C were also combined (serotype 6A/C), since, at the time of the study, antisera-based serotyping could not distinguish between the two. Pneumococcal serotype 6D has also been described recently: this serotype may be identified as 6B using currently available antisera [14], [15]. There are no data regarding prevalence of this serotype in the study population, although given the absence of PCV use, carriage this serotype was unlikely to be common and thus antisera 6B isolates have been reported as such [16], [17]. Although commonly carried in the nasopharynx, non-typeable pneumococci were excluded from these comparisons, since the latex sweep method has no confirmatory test of pneumococcal identity except the presence of capsule.

### Ethics Statement

Written informed consent was obtained from the mothers of all study infants prior to study enrolment. Ethical approval for this study was granted by the ethics committees of the Faculty of Tropical Medicine, Mahidol University, Thailand (MUTM-2009-306) and Oxford University, UK (OXTREC-031-06).

## Results

### Pneumococcal Colonisation in “routine” Follow-up Group Infants

Three hundred and sixty four infants from the “routine” follow-up group had a complete 24 month NPS specimen set. A total of 8,736 swabs were cultured and latex sweep serotyping done (Figure 1). Typeable pneumococci were identified in 65.3% (5,699/8,736) of specimens. Multiple pneumococcal serotypes were found in 734 NPS specimens (12.9% of specimens from which pneumococci were cultured). In swabs with more than one serotype, the median number of serotypes detected was two and the maximum was four.

### Serotype Distribution

**Figure 2:**
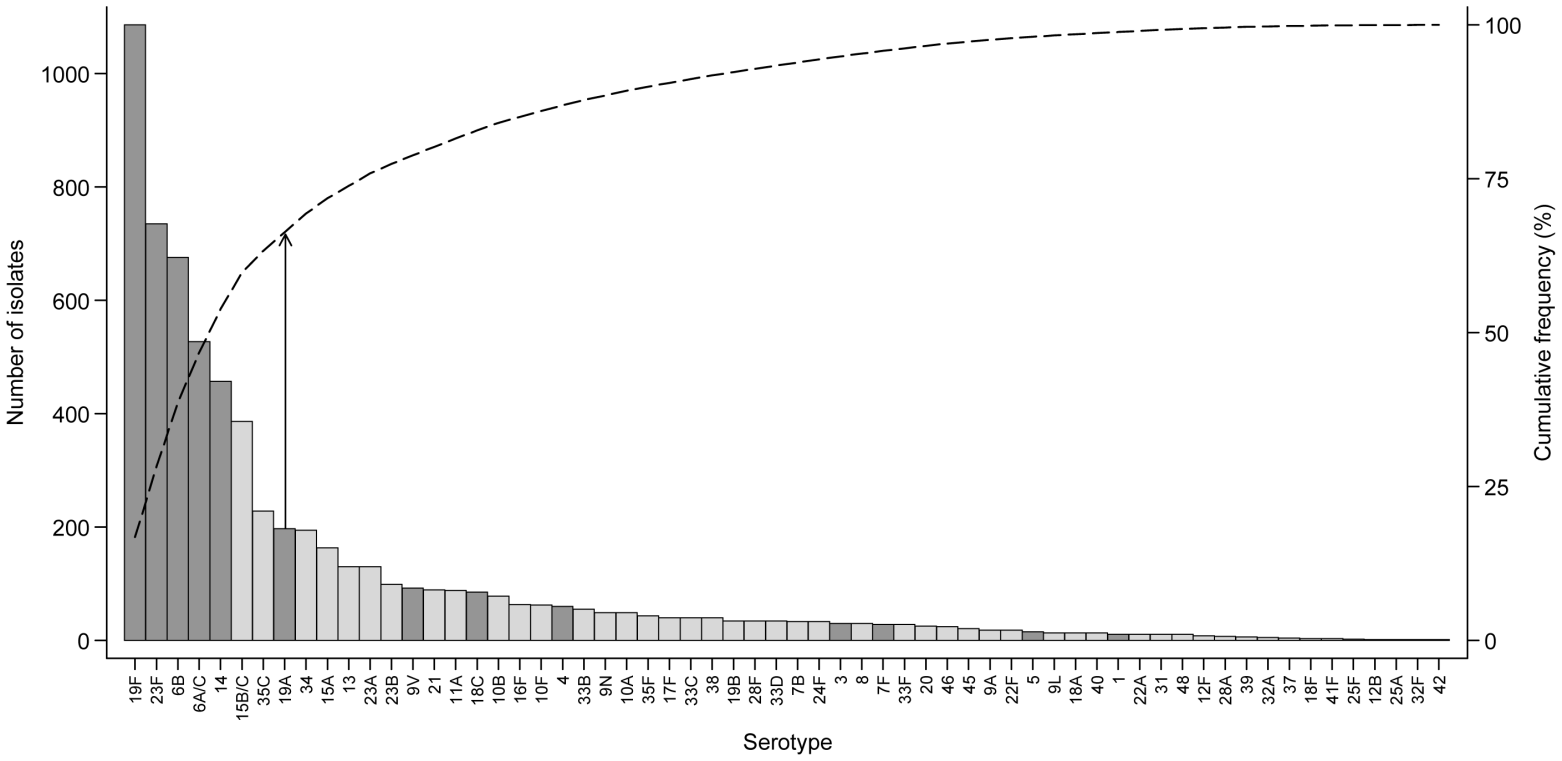
Infant carriage pneumococcal serotype distribution, by latex sweep serotyping. Results of culture of 8,736 swabs from 364 infants. The bars indicate the number of isolates of each serotype and the dashed lines indicate the cumulative frequency (cumulative frequency of 67% is indicated by the vertical arrows). Dark grey bars highlight PCV13 serotypes.

A total of 62 serotypes were identified and eight of these (19F, 23F, 6B, 6A/C, 14, 15B/C, 35C, 19A) accounted for two-thirds of all pneumococci detected (Figure 2). Almost two thirds (61.8%; 3,999/6,471) of the pneumococci detected were PCV13 serotypes and 42.4% (3,704/8,736) NPS contained at least one PCV13 serotype.

The ten most prevalent serotypes in these infants were identical to those in the 234 infants from the “immunology” follow-up group (excluding NT pneumococci), although the rank order varied [8].

### Agreement with WHO Culture within Specimens

One thousand one hundred and seven (12.7%) of the NPS specimens from these 364 infants had previously been cultured using the WHO protocol. At least one typeable pneumococcus was identified in 68.6% of NPS specimens by WHO culture and 64.0% by sweep method (p<0.001). 9.3% of specimens contained multiple serotypes by sweep method compared with 3.1% by WHO method (p<0.001). However, there were no differences in the proportion of specimens containing at least one PCV13 serotype or non-vaccine serotype (NVT) pneumococcus by method (Table 1).

**Table 1:** Comparison of NPS culture and serotyping results by processing method.

	WHO method	Sweep method	p-value
	N (%)	N (%)	
At least one typeable pneumococcus detected	759 (68.6)	708 (64.0)	<0.001
Multiple pneumococcal serotypes detected	34 (3.1)	103 (9.3)	<0.001
PCV13 serotype(s) detected	487 (44.0)	473 (42.7)	0.1
NVT serotype(s) detected	291 (26.3)	280 (25.3)	0.2
Identical serotype if detected by WHO culture	–	85.0%	–
Identical serogroup if detected by WHO culture	–	86.4%	–

1,107 nasopharyngeal swabs evaluated by both methods.

In swabs containing at least one typeable pneumococcus by the WHO method, an identical serotype was identified in 85.0% (645/759) by the sweep method. Relaxed to agreement at the serogroup/type level, this proportion increased only slightly to 86.4% (656/759). Where both methods identified at least a single typeable pneumococcus, agreement was 91.2% (645/707; serotype level) and 92.8% (565/707; serogroup/type level), perhaps indicating that low density pneumococcal colonisation was missed by the latex sweep method. Serotype agreement between methods was lowest in specimens when there was light colonisation (defined as 1+) on the WHO culture plate (Table 2). Although not statistically significant at the serotype level, the likelihood of agreement at serogroup/type level was significantly higher if colonisation density was 2+ or greater (OR 1.65, 95% CI 1.06–2.57, p = 0.03).

**Table 2:** Relationship between colonisation density and agreement between WHO culture and latex sweep result.

Colonisation density(WHO culture plate)	Serotype agreementN (%)	Total
1+	311 (80.4)	387
2+	170 (89.5)	190
3+	96 (92.3)	104
4+	68 (87.2)	78
Total	648 (85.0)	759

### Comparison of Pneumococcal Colonisation Dynamics: Infant Swabs Cultured by WHO or Latex Sweep Methods

One hundred “immunology” follow-up group infants whose NPS had been cultured by the WHO method were matched 1∶1 with “routine” follow-up group infants whose swabs were processed by the latex sweep method. All infants contributed 24 monthly swabs and all were colonised by pneumococci at least once.

**Figure 3:**
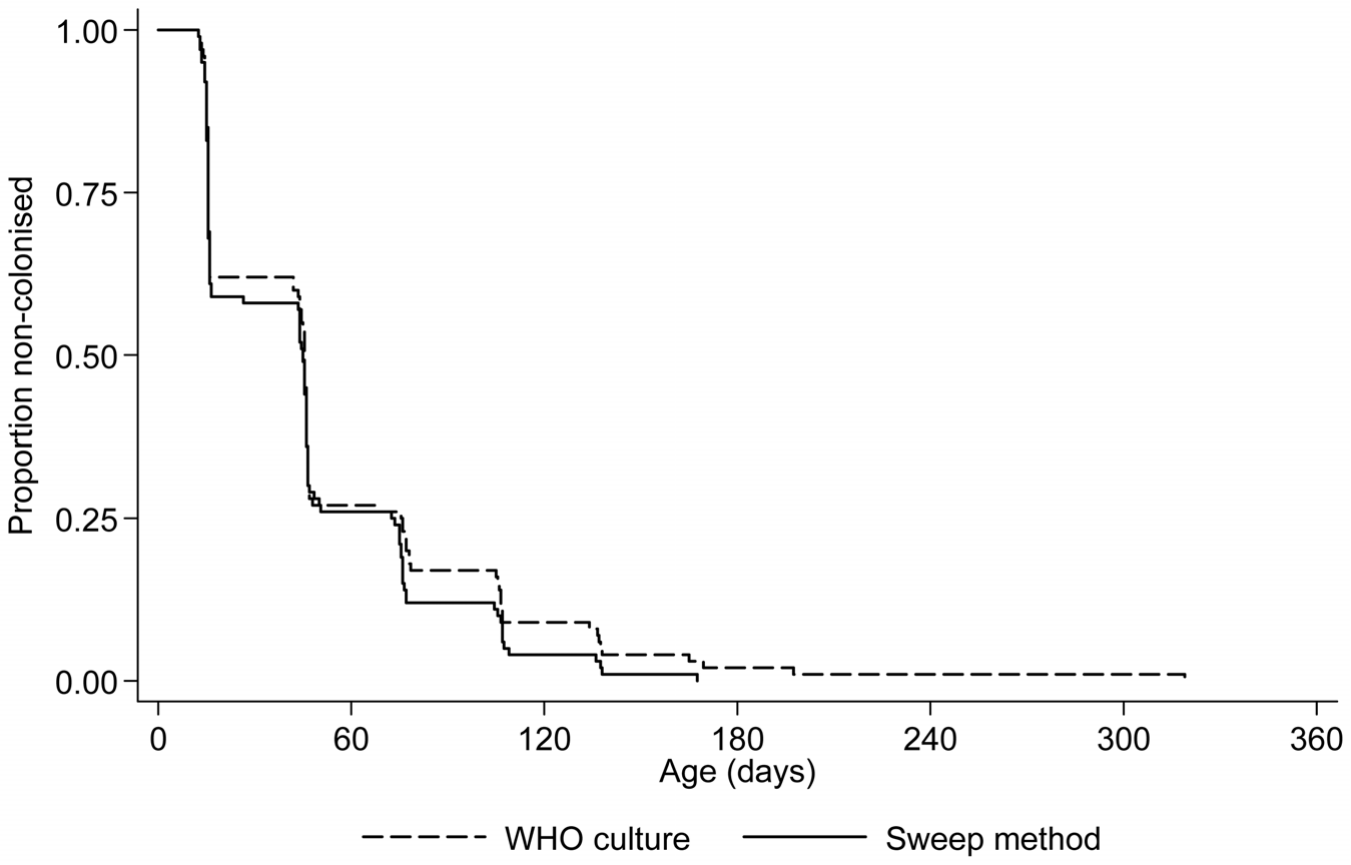
Age at first pneumococcal acquisition, stratified by NPS culture/serotyping method. 100 infants were included in each group.

**Figure 4:**
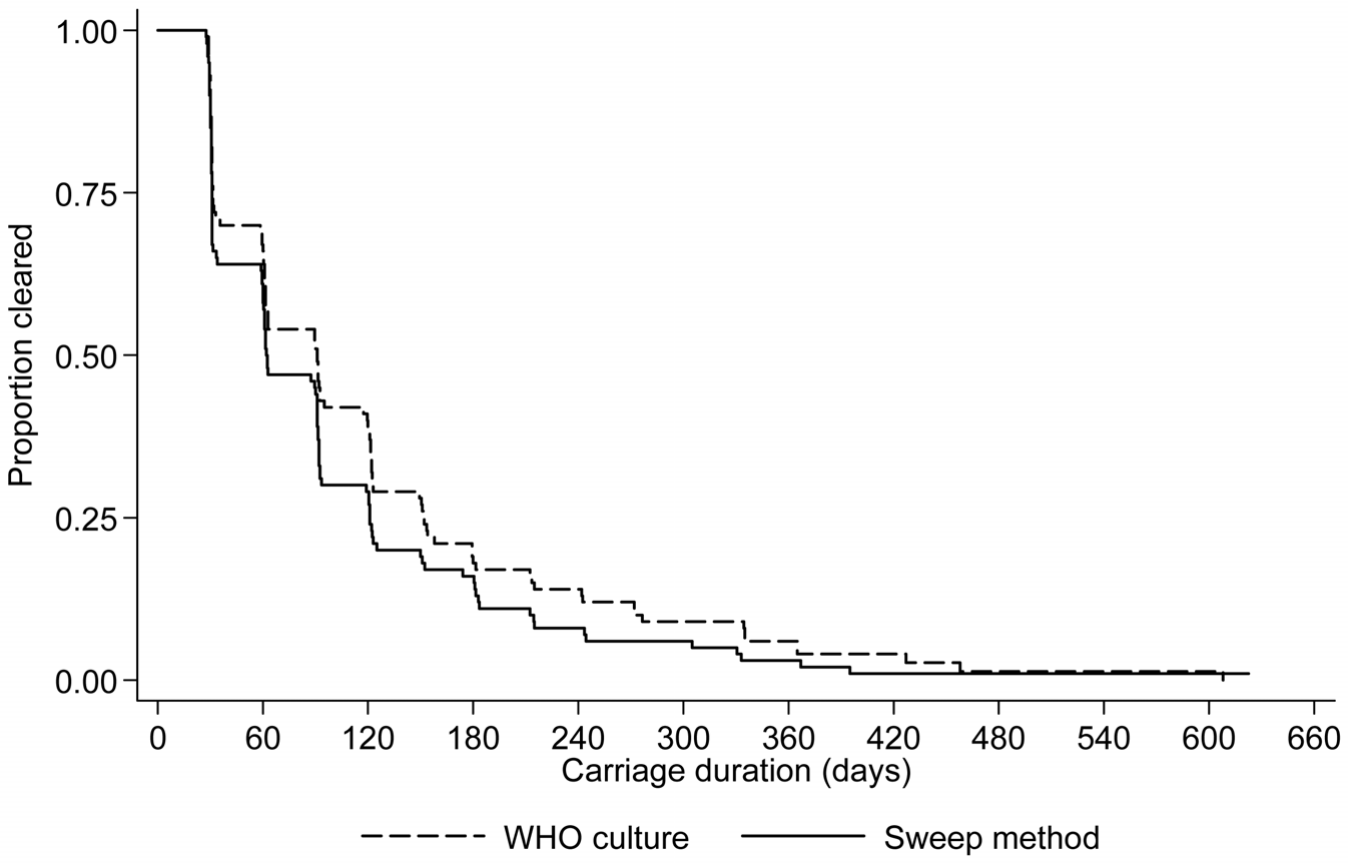
Duration of first pneumococcal carriage episode, stratified by NPS culture/serotyping method. 100 infants were included in each group.

In both groups, the median age at first pneumococcal acquisition was 46 days (p = 0.6; Figure 3). Considering individual serotype acquisition rates, there were no significant differences in rates of acquisition of commonly carried PCV13 serotypes. However, the non-vaccine serotype acquisition rate was higher in infants whose swabs were processed by the latex sweep method (Table 3). Durations of first carriage episodes, without stratification by serotype, were similar in both groups (“immunology”/WHO: median 90 days (IQR 31–151); “routine”/latex sweep: 62 days (IQR 31–121); p = 0.2; Figure 4).

**Table 3:** Individual pneumococcal serotype acquisition rates, by NPS culture/serotyping method.

Serotype	Swabs cultured by WHO method			Swabs cultured by sweep method			p-value
	N	Acq rate^a^	95% CI	N	Acq rate^a^	95% CI	
**19F**	56	0.0012	(0.0009–0.0016)	59	0.0012	(0.0010–0.0016)	0.9
**23F**	51	0.0010	(0.0008–0.0013)	44	0.0008	(0.0006–0.0011)	0.3
**6B**	49	0.0009	(0.0007–0.0012)	50	0.0009	(0.0007–0.0012)	0.9
**6A/C**	47	0.0008	(0.0006–0.0011)	48	0.0009	(0.0007–0.0012)	0.6
**15B/C**	36	0.0006	(0.0004–0.0008)	51	0.0009	(0.0007–0.0012)	0.03
**14**	35	0.0006	(0.0004–0.0008)	37	0.0006	(0.0004–0.0009)	0.8
**19A**	25	0.0004	(0.0003–0.0006)	32	0.0005	(0.0004–0.0007)	0.3
**34**	22	0.0003	(0.0002–0.0005)	32	0.0005	(0.0004–0.0008)	0.1
**15A**	15	0.0002	(0.0001–0.0004)	20	0.0003	(0.0002–0.0005)	0.4
**35C**	12	0.0002	(0.0001–0.0003)	29	0.0005	(0.0003–0.0007)	0.002
**PCV13**	100	0.0058	(0.0048–0.0071)	100	0.0070	(0.0057–0.0085)	0.2
**NVT** b	92	0.0047	(0.0038–0.0057)	98	0.0071	(0.0058–0.0086)	0.04
**All** b	100	0.0150	(0.0123–0.0183)	100	0.0165	(0.0135–0.0200)	0.6

100 infants were included in each group; each serotype considered independently.

a Acquisition rate per day.

b Non-typeable pneumococcal colonisation excluded.

Infants with swabs processed using the latex sweep method had a greater number of pneumococcal serotype carriage episodes than those whose swabs were cultured using the WHO method (mean 8.4 vs. 7.5, p = 0.03). There were no differences in the number of PCV13 or NVT serotype carriage episodes between the groups (PCV13 serotypes accounted for 51.4% (“immunology”/WHO) and 54.0% (“routine”/sweep) carriage episodes, p = 0.3).

## Discussion

This study is the first to look at the implementation of more sensitive techniques for multiple pneumococcal serotype isolation in the field and the first to show that, despite the greater sensitivity of the sweep assay, serotype distributions and serotype colonisation dynamics reported in carriage studies are unlikely to change significantly if the new methods are used. However, the work confirms the preliminary finding that the latex sweep serotyping method is significantly more sensitive than WHO culture followed by serotyping of morphologically distinct colonies for detection of multiple pneumococcal serotype colonisation: an important finding for studies working to determine the interactions between pneumococcal serotypes, vaccines, and other members of the nasopharyngeal microbiome [6]. It also demonstrates the feasibility of processing large numbers of NPS specimens using the latex sweep method: over 8,000 swabs were turned around in less than 12 months. Currently described alternative methods to improve multiple serotype colonisation are unlikely to be able to manage such numbers, especially when factoring in the costs of molecular assays or those phenotypic assays requiring significant volumes of pneumococcal antisera [18]–[20]. Increased throughput did not negatively impact on serotype agreement between two methods: a common serotype was found in 92.8% swabs. In our initial comparison of serotyping methods, which included microarray, a common serogroup/type was identified in 95.2% specimens [6]. This is an important finding, especially since recent work has determined that molecular serotyping directly from broth enrichment cultures may identify *cps* locus genes present in non-pneumococcal streptococci resulting in false positive “pneumococcal” serotype detections [21].

Given the high prevalence of carriage of non-typeable pneumococci, improved confirmation of colonisation by these organisms would be helpful [22]. Although non-specific weak agglutination by latex can presumptively identify these organisms, without a confirmatory test of pneumococcal identity their carriage prevalence may be over-estimated by the latex sweep method. The finding that the latex sweep method was significantly less sensitive at serotype detection from swabs with lower pneumococcal density is also a problem and further work to improve this is required, especially for the technique to be successfully employed in situations where low colonisation density may be anticipated [23], [24]. This is relevant since studies of colonisation in immunised infants and also adults are of considerable importance in monitoring for changing in serotype carriage prevalence following introduction of conjugate vaccines [4]. One possible solution might be to continue to use the WHO methodology to identify the dominant pneumococcal serotype in specimens low density carriage (i.e. 1+ growth) and use the latex sweep method for higher density carriage specimens. The reduced sensitivity of low density pneumococcal colonisation detection by the latex sweep method may explain why the overall prevalence of multiple serotype colonisation reported here (12.9% (734/5,699) of swabs from which pneumococci were cultured in the “routine” cohort infants) was lower than in some other studies. For example, in Papua New Guinea, Gratten et al. identified multiple serotypes in 29.5% of children carrying pneumococci [25]. However, the three-fold increase in multiple serotype detection (9.3% vs. 3.1%) when comparing sweep results to WHO culture/serotyping in the same specimen selection from Maela infants is a reasonably large increment. In the current study, WHO culture equated to serotyping all morphologically discrete pneumococci. Comparing this strategy to selection of four random colonies for characterisation, Hare et al. identified an additional 3% (14% vs. 17%) of NPS specimens containing multiple pneumococcal serotypes in Australian Aboriginal children: put another way, random colony selection resulted in a 1.2-fold increase in multiple colonisation detection over the WHO approach [26]. The additional freeze-thaw cycle, required in the current study for the latex sweep work on NPS previously cultured by the WHO method, may have impacted on the sensitivity analyses presented here. However, a previous comparison of NPS-STGG specimens cultured directly and after a freeze-thaw cycle demonstrated 98% concordance in pneumococcal detection [27].

Infant serotype distribution was not affected by culture/serotyping methodology in this study. Excluding non-typeable pneumococci, the top ten ranking serotypes in infant swabs processed by WHO method or latex sweep were identical, with a small number of serotypes accounting for the majority of pneumococci identified. This finding agrees with the work of Charalambous and colleagues, who demonstrated that population-level characteristics of colonising pneumococci could be adequately described by characterisation of a single colony per specimen [28]. Age at first pneumococcal acquisition was identical, and durations of first pneumococcal carriage were similar, in infants whose swabs were processed by either method. Estimates for NVT acquisition rates were higher (both overall and for serotypes 15B/C and 35C individually) in infant whose swabs were processed by latex sweep. These infants were also determined to have a greater number of discrete serotype colonisation episodes compare to infants whose swabs were cultured by the WHO method. Sweep serotyping was not significantly more likely detect NVT pneumococcal colonisation overall. However, a limitation of this study was that differences observed in colonisation dynamics in the case-control component may reflect unaccounted for, and potentially important, environmental factors [29]. Infants were matched by gender and month of birth, but household differences were not measured.

In conclusion, this study has demonstrated accuracy and feasibility of the latex sweep serotyping method for detection of pneumococcal colonisation in longitudinal infant studies. Further work to improve detection of colonisation by non-typeable organisms and pneumococci at low density is required.
